# Factors associated with COVID-19 vaccine hesitancy among healthcare workers in Cameroon and Nigeria: a web-based cross-sectional study

**DOI:** 10.1093/inthealth/ihad013

**Published:** 2023-03-11

**Authors:** Jerry Brown Aseneh, Valirie Ndip Agbor, Benjamin Momo Kadia, Elvis Anyaehiechukwu Okolie, Chinelo Janefrances Ofomata, Christie Linonge Etombi, Domin Sone M Ekaney, Yvonne Walburga Joko Fru

**Affiliations:** Department of Health Research, Health Education and Research Organization (HERO), Buea, 154, Cameroon; Ecole de Santé Publique, Université Libre de Bruxelles, Brussel, 1070, Belgium; Department of Health Research, Health Education and Research Organization (HERO), Buea, 154, Cameroon; Clinical Trial Service Unit and Epidemiological Studies Unit (CTSU), Nuffield Department of Population Health, University of Oxford, Oxford, OX3 7LF, UK; Department of Health Research, Health Education and Research Organization (HERO), Buea, 154, Cameroon; Department of Clinical Sciences, Liverpool School of Tropical Medicine, Liverpool, L3 5QA, UK; Department of Public Health, School of Health and Life Sciences, Teesside University, Middlesbrough, TS1 3BX, UK; Nuffield Centre for International Health and Development, University of Leeds, Leeds, LS2 9JT, UK; Department of Health Research, Health Education and Research Organization (HERO), Buea, 154, Cameroon; Department of Health Research, Health Education and Research Organization (HERO), Buea, 154, Cameroon; Cancer and Epidemiology Unit (CEU), Nuffield Department of Population Health, University of Oxford, Oxford, OX3 7LF, UK; The African Cancer Registry Network, INCTR African Registry Programme, Oxford, OX2 7HT, UK

**Keywords:** acceptability, acceptance, COVID-19, health workers, hesitancy, sub-Saharan Africa, vaccine

## Abstract

**Background:**

This study investigated the determinants of coronavirus disease 2019 (COVID-19) vaccine hesitancy among healthcare workers (HCWs) in Cameroon and Nigeria.

**Methods:**

This analytic cross-sectional study was conducted from May to June 2021, including consenting HCWs aged ≥18 y identified using snowball sampling. Vaccine hesitancy was defined as indecisiveness or unwillingness to receive the COVID-19 vaccine. Multilevel logistic regression yielded adjusted ORs (aORs) for vaccine hesitancy.

**Results:**

We included a total of 598 (about 60% women) participants. Little or no trust in the approved COVID-19 vaccines (aOR=2.28, 95% CI 1.24 to 4.20), lower perception of the importance of the vaccine on their personal health (5.26, 2.38 to 11.6), greater concerns about vaccine-related adverse effects (3.45, 1.83 to 6.47) and uncertainty about colleagues’ acceptability of the vaccine (2.98, 1.62 to 5.48) were associated with higher odds of vaccine hesitancy. In addition, participants with chronic disease (aOR=0.34, 95% CI 0.12 to 0.97) and higher levels of concerns about getting COVID-19 (0.40, 0.18 to 0.87) were less likely to be hesitant to receive the COVID-19 vaccine.

**Conclusions:**

COVID-19 vaccine hesitancy among HCWs in this study was high and broadly determined by the perceived risk of COVID-19 and COVID-19 vaccines on personal health, mistrust in COVID-19 vaccines and uncertainty about colleagues’ vaccine acceptability.

## Introduction

Coronavirus disease 2019 (COVID-19), caused by the severe acute respiratory syndrome coronavirus-2 (SARS-CoV-2) virus, was identified at the end of 2019 and has claimed >5 million lives and >270 million confirmed cases.^1^ The prevalence and mortality of COVID-19 vary substantially across populations owing, in part, to the degree of adherence to containment measures, the availability of reliable diagnostics and reporting systems, demographics, climate and environmental factors, genetics and immunologic variations.^2^ The WHO's Strategic Preparedness and Response Plan 2021 sought to curb the burden of COVID-19 by strengthening national health systems to prevent, diagnose and treat COVID-19.^3^ COVID-19 immunization is crucial to limit the spread of the SARS-CoV-2 virus and the severity of COVID-19, thereby reducing disease-related disability and death. Additionally, large-scale population immunity (herd immunity) is necessary to prevent vulnerable populations who, for some reason, are not eligible for the vaccine. Herd immunity has helped eradicate deadly infectious diseases like smallpox.^4^ The COVID-19 Vaccine Global Access (COVAX) facility works towards ensuring equitable access to safe and effective COVID-19 vaccines globally.^5^ As of November 2021, 7.8 billion vaccine doses had been given globally.^6^ About 227 million vaccine doses had been supplied to the African population. However, high rates of COVID-19 vaccine hesitancy have hampered efforts towards achieving higher vaccination coverage despite improvements in the availability and accessibility of vaccines.^7,8^ By February 2022, only about 6.5% and 11.9% of the general population in Cameroon and Nigeria, respectively, had received at least one COVID-19 vaccine.^9^ The rate of vaccine acceptance in the general population remained heterogenous across Africa; it ranged from 6.9% to 97.9%.^10^ Vaccine safety and side effects, lack of trust in pharmaceutical industries and misinformation or conflicting information from the media were factors associated with vaccine hesitancy.^10^

Healthcare workers (HCWs) are a priority population in the current COVID-19 vaccination strategy because of increased workplace exposure to COVID-19.^11^ High vaccination coverage among HCWs is crucial in preventing severe COVID-19, reducing transmission to patients and close contacts and ensuring that healthcare systems are fully operational in such difficult moments.^12^ Moreover, HCWs play a role in instilling confidence in the general population about vaccine safety and efficacy.^13^ Nevertheless, the rate of COVID-19 vaccine acceptability and uptake among HCWs in Africa was heterogenous and quite low in some settings, despite the efforts of COVAX to improve the availability of the vaccine on the continent.^5^ It ranged from 24.3% to 90.1%.^14–30^ By November 2021, only one in four African HCWs was fully vaccinated against COVID-19,^12^ and only 300 000 (about 18%) of its 1.6 million health workers had been vaccinated in Nigeria.^12^

The determinants of COVID-19 vaccine hesitancy among HCWs include concerns about the vaccine's safety and efficacy and distrust in government and public health regulatory authorities.^23,31^ Understanding and addressing the drivers of COVID-19 vaccine hesitancy among HCWs in Africa is pivotal to improving vaccine uptake and curbing the burden of COVID-19 in Africa. Therefore, this study was performed to investigate the factors associated with COVID-19 vaccine-hesitant attitudes among HCWs in Cameroon and Nigeria. This is necessary to shed more light on understanding COVID-19 vaccine hesitancy, buttress reoccurring determinants of vaccine hesitancy and aid in framing effective strategies in addressing them.

## Methods

### Study design, period and setting

This was a web-based cross-sectional study conducted from 1 May to 31 July 2021. As of 2021, Nigeria was the most populous country in Africa, with a population of 221 million, while Cameroon had a population of 28 million.^32^ Nigeria had a health worker-to-population ratio of about 3.8 medical doctors per 10 000 population and 15 nursing and midwifery personnel per 10 000 population in 2019.^33^ Meanwhile, Cameroon had a health worker-to-population ratio of about 1.3 medical doctors per 10 000 population and 3.6 nursing and midwifery personnel per 10 000 population in 2018.^33^

During 15–22 September 2021, there 2974 new cases of COVID-19 and 83 COVID-19–related deaths in Cameroon. The case fatality rate was 1.7%. Because of the lack of vaccines for widespread immunization campaigns, only 1.2% of the target population (all people aged ≥18 y) were vaccinated by October 2021. This low vaccination rate was attributed to the low number in the workforce and the reluctance of the population to receive the vaccine.^34^ COVID-19 vaccination commenced in March and April 2021 in Nigeria and Cameroon, respectively. A total of 42.6 and 7.3 doses per 100 population were administered in Nigeria and Cameroon, respectively.^6^

### Participants

We recruited consenting HCWs (medical doctors, nurses, medical laboratory technicians, midwives, paramedics, nurse assistants, community health workers and administrative staff who are directly and indirectly in contact with patients) aged ≥18 y practicing in Cameroon or Nigeria.

### Sample size calculation and sampling

Cochran's formula was used to calculate the minimum acceptable sample size (n) for a margin of error (d) of 5% and a standard normal deviate of 1.96. We estimated that about 50% of HCWs would be hesitant to receive the COVID-19 vaccine. The estimate of 50% was arbitrarily chosen because there were no estimates of the prevalence of COVID-19 hesitancy in similar settings at the time of this study. Given the low vaccine uptake and our appraisal of what HCWs in these countries thought about the vaccine, we estimated that at least 50% of HCWs would be hesitant to receive the vaccine.


\begin{equation*}{\mathrm{n}} = {{\mathrm{z}}}^2*{\mathrm{p}}\left( {1 - {\mathrm{p}}} \right)/{{\mathrm{d}}}^2 = 384.\end{equation*}

Participants were recruited electronically using a snowballing technique.

### Data collection

A secured online Google Form was designed as a self-administered version of the standardized questionnaire by the WHO to assess the drivers of COVID-19 vaccine acceptability in adults.^35^ The questionnaire was pretested and disseminated through existing groups in messaging applications (WhatsApp and Telegram Forums) created for HCWs in Nigeria or Cameroon and social media platforms (Facebook, Twitter and LinkedIn). Data collectors (comprising medical doctors, nurses, pharmacists and dentists) disseminated the online survey link. HCWs were encouraged to share the link with their colleagues and other relevant groups. We adopted this approach to ensure physical distancing, limiting the transmission of the virus.

### Measurement and variables

The primary outcome was COVID-19 vaccine hesitancy. COVID-19 vaccine hesitancy was assessed using the following questions: Have you received any COVID-19 vaccine? (Yes or no); and if you have not received a COVID-19 vaccine, do you intend to take the vaccine if it were available? (Yes, no and not sure). Participants were considered hesitant to the COVID-19 vaccine if they had not received any dose of the vaccine and were unwilling or unsure about getting it despite its availability.^36^

The independent variables included:

- **Sociodemographic data**

Age (in y), gender, current professional role (e.g. medical doctor, dentist, pharmacist, nurse or administrative staff) and area of work facility (urban vs rural).

- **Medical history**

Participants were requested to report any history of chronic disease (yes or no). In addition, history of COVID-19 was assessed using the following questions: To your knowledge, do you have or have you had COVID-19? (Yes or no) and if yes, was COVID-19 confirmed by a test? (Yes or no).

- **COVID-19 risk perception, and benefits and safety of COVID-19 vaccines**

Participants’ perception of the risk of COVID-19 was assessed by asking about concerns about themselves, their close family or friends and patients getting COVID-19 on a four-point Likert scale (not at all, a little, moderately and very concerned). In addition, we assessed: participants’ perception of the benefits of the COVID-19 vaccine to their health and others in their community (not at all to very important); participants’ perception of harm related to the vaccine (participants’ perception of safety of the vaccine to their health [not at all to very safe] and concerns about developing serious adverse reactions to the vaccine [not at all to very concerned]); how much the participant wanted the vaccine (not at all to very much or had received the vaccine); whether they were willing to recommend the vaccine to eligible individuals (yes, no or not sure); and participants’ confidence in answering patients’ questions related to the COVID-19 vaccine (not at all to very confident).

- **Social factors related to the COVID-19 vaccine uptake**

Participants were asked whether they needed permission to receive the COVID-19 vaccine (yes or no) and if they thought that most of their close friends and family members, community or religious leaders and colleagues would like to receive the vaccine (yes, no or not sure). In addition, participants’ level of trust in the national ministry of health (MoH) was assessed on a four-point Likert scale (not at all to very much). Finally, participants were asked if they had heard anything bad about the vaccine (yes or no).

- **Others**

Participants were asked if they received poor treatment during the COVID-19 period because of their profession (yes, no or not sure).

### Data management and statistical analysis

Stata 17 software (StataCorp, College Station, TX, USA) and R programming software (version 3.5.1, 2019, The R Foundation for Statistical Computing, Vienna, Austria) were used for data analysis. Quantitative variables were summarized using the mean (and SD) or median (with IQR) depending on their distribution. Categorical variables were summarized using frequencies or percentages, and the 95% CI for the prevalence of COVID-19 vaccine hesitancy.

Responses of participants from Nigeria are likely to be more similar than Cameroon due to similar demographics, introducing clustering in our data. However, clustering violates the assumption of data independence and increases the likelihood of type I error.^37,38^ We evaluated model non-dependence using the likelihood ratio (LR) test by allowing model intercept to vary randomly across countries. Due to significant evidence for model dependence, we fitted multilevel logistic regression models to evaluate factors independently associated with COVID-19 vaccine hesitancy. Variables with p<0.25 on univariate analysis^39^ and variables reported to be associated with vaccine hesitancy (or acceptability) in the published literature were considered for inclusion in the multivariable analysis. Independent variables were sequentially included in the multivariable model. The LR test was used to assess model fit. Only variables that improved model fit were retained in the final multivariable model.

We assessed departures from linearity in ordinal and continuous variables using the LR test. Ordinal variables were modeled to evaluate linear trends without evidence for deviations from linearity. By contrast, the p-value from the LR test for heterogeneity was used to assess statistical significance in nominal and ordinal variables (where there was evidence of departure from linearity). We preferred the LR over the Wald test for inference as it is more powerful and robust. Missing data were addressed using simple imputation of the mode or mean, where appropriate. Two-tailed p<0.05 was considered statistically significant.

## Results

### Characteristics of the study population

**Figure 1.**
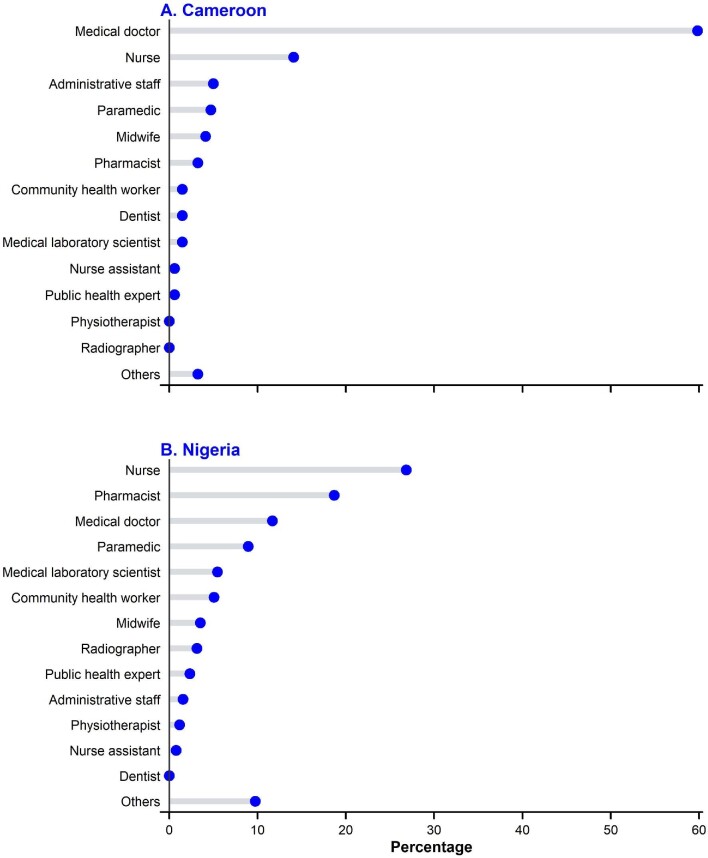
Job specificity of healthcare workers in (A) Cameroon and (B) Nigeria.

Of the 598 healthcare workers who participated in our study, 257 (43%) were from Nigeria. The mean age of the participants was 29.4 (SD=5.9) y and was similar between participants from both countries. In addition, most participants were female (55.9%), worked in urban settings (78.8%), had no chronic disease (92.5%) and had no previous or current COVID-19 (73.1%) (Table 1). Meanwhile, only 127 (21.2%) had received any dose of a COVID-19 vaccine. Most of the participants consisted of medical doctors, nurses, midwives, pharmacists and other hospital staff, like administrative staff (Figure 1).

**Table 1.** Sociodemographic characteristics of the study population

Characteristic	Cameroon	Nigeria	Total
	N=341	N=257	N=598
Age (y)	29.3 (5.2)	29.5 (6.7)	29.4 (5.9)
Gender			
Female	177 (51.9)	157 (61.1)	334 (55.9)
Male	164 (48.1)	100 (38.9)	264 (44.1)
Area of work			
Rural area	84 (24.6)	43 (16.7)	127 (21.2)
Urban area	257 (75.4)	214 (83.3)	471 (78.8)
Known chronic disease			
No	313 (91.8)	240 (93.4)	553 (92.5)
Yes	28 (8.2)	14 (5.4)	42 (7.0)
Missing	0 (0.0)	3 (1.2)	3 (0.5)
Previous or current COVID-19			
No	215 (63.0)	222 (86.4)	437 (73.1)
Yes, confirmed	73 (21.4)	14 (5.4)	87 (14.5)
Yes, not confirmed	53 (15.5)	21 (8.2)	74 (12.4)
Received a COVID-19 vaccine			
No	277 (81.2)	159 (61.9)	436 (72.9)
Yes	59 (17.3)	68 (26.5)	127 (21.2)
Missing	5 (1.5)	30 (11.7)	35 (5.9)
Treated poorly during the pandemic due to profession			
No	270 (79.2)	180 (70.0)	450 (75.3)
Yes	71 (20.8)	77 (30.0)	148 (24.7)

Abbreviations: COVID-19, coronavirus disease 2019; N, frequency.

### Risk perception on COVID-19

About 50% of the participants were moderately or very concerned about getting COVID-19 (Table 2). In addition, approximately 68% and 71% of the participants had at least moderate concerns about their friends or families and patients developing COVID-19, respectively.

**Table 2.** COVID-19 risk perception by study participants

Characteristic	Cameroon	Nigeria	Total
	N=341	N=257	N=598
Concerned about getting COVID-19			
Not at all concerned	78 (22.9)	51 (19.8)	129 (21.6)
A little concerned	100 (29.3)	60 (23.3)	160 (26.8)
Moderately concerned	83 (24.3)	61 (23.7)	144 (24.1)
Very concerned	80 (23.5)	85 (33.1)	165 (27.6)
Concerned about family or friends getting COVID-19			
Not at all concerned	44 (12.9)	36 (14.0)	80 (13.4)
A little concerned	60 (17.6)	49 (19.1)	109 (18.2)
Moderately concerned	75 (22.0)	48 (18.7)	123 (20.6)
Very concerned	162 (47.5)	124 (48.2)	286 (47.8)
Concerned about patients getting COVID-19			
Not at all concerned	40 (11.7)	36 (14.0)	76 (12.7)
A little concerned	56 (16.4)	40 (15.6)	96 (16.1)
Moderately concerned	72 (21.1)	48 (18.7)	120 (20.1)
Very concerned	173 (50.7)	132 (51.4)	305 (51.0)
Missing	0 (0.0)	1 (0.4)	1 (0.2)

Abbreviations: COVID-19, coronavirus disease 2019; N, frequency.

### Perception of the benefit and harm of COVID-19

Of the 598 participants, 50% of the respondents had little or no trust in the approved COVID-19 vaccines, 65% were moderately or very concerned about vaccine-related adverse reactions and 61% had little or no trust in the MoH (Table 3). Nevertheless, more than one-half of the participants perceived the COVID-19 vaccine to be moderately or very important to personal health (58.7%) and protect the community from COVID-19 (62.5%). About 66.7% of the participants were willing to recommend the vaccine to eligible persons. Only 29.3% of the participants were sure their colleagues would get the vaccine. In addition, 26.1% of the participants were certain that their community or religious leaders would approve of getting the vaccine. Similarly, 26.4% were sure that their friends and families would support receiving the vaccine.

**Table 3.** Perception of the benefit and harm of COVID-19 vaccine

Characteristics	Cameroon	Nigeria	Total
	N=341	N=257	N=598
Intention to take COVID-19 vaccine			
Taken the vaccine	59 (17.3)	68 (26.5)	127 (21.2)
Not taken the vaccine but intend to	88 (25.8)	80 (31.1)	168 (28.1)
Unsure about taking vaccine	105 (30.8)	61 (23.7)	166 (27.8)
Do not intend to take vaccine	89 (26.1)	48 (18.7)	137 (22.9)
Trust in approved COVID-19 vaccines			
Not at all	89 (26.1)	40 (15.6)	129 (21.6)
A little	89 (26.1)	90 (35.0)	179 (29.9)
Moderately	124 (36.4)	91 (35.4)	215 (36.0)
Very much	37 (10.9)	35 (13.6)	72 (12.0)
Missing	2 (0.6)	1 (0.4)	3 (0.5)
Concerns about vaccine-related adverse reaction			
Not at all concerned	34 (10.0)	21 (8.2)	55 (9.2)
A little concerned	87 (25.5)	60 (23.3)	147 (24.6)
Moderately concerned	76 (22.3)	64 (24.9)	140 (23.4)
Very concerned	138 (40.5)	112 (43.6)	250 (41.8)
Missing	6 (1.8)	0 (0.0)	6 (1.0)
Impression about importance of COVID-19 vaccine on personal health			
Not at all important	78 (22.9)	34 (13.2)	112 (18.7)
A little important	77 (22.6)	54 (21.0)	131 (21.9)
Moderately important	91 (26.7)	63 (24.5)	154 (25.8)
Very important	92 (27.0)	105 (40.9)	197 (32.9)
Missing	3 (0.9)	1 (0.4)	4 (0.7)
Getting the COVID-19 vaccines protects the community from COVID-19			
Not at all	69 (20.2)	35 (13.6)	104 (17.4)
A little	75 (22.0)	41 (16.0)	116 (19.4)
Moderately	96 (28.2)	74 (28.8)	170 (28.4)
Very much	97 (28.4)	107 (41.6)	204 (34.1)
Missing	4 (1.2)	0 (0.0)	4 (0.7)
Impression about safety of COVID-19 vaccine on personal health			
Not at all safe	75 (22.0)	35 (13.6)	110 (18.4)
A little safe	93 (27.3)	44 (17.1)	137 (22.9)
Moderately safe	118 (34.6)	105 (40.9)	223 (37.3)
Very safe	50 (14.7)	71 (27.6)	121 (20.2)
How much participant wants the vaccine			
Not at all	116 (34.0)	56 (21.8)	172 (28.8)
A little	76 (22.3)	51 (19.8)	127 (21.2)
Moderately	79 (23.2)	65 (25.3)	144 (24.1)
Very much/Received the vaccine	63 (18.5)	84 (32.7)	147 (24.6)
Missing	7 (2.1)	1 (0.4)	8 (1.3)
Willing to recommend COVID-19 vaccine to eligible persons			
Yes	217 (63.6)	182 (70.8)	399 (66.7)
Not sure	82 (24.0)	47 (18.3)	129 (21.6)
No	38 (11.1)	28 (10.9)	66 (11.0)
Missing	4 (1.2)	0 (0.0)	4 (0.7)
Friends and family's opinion about getting the vaccine			
Disapprove	164 (48.1)	68 (26.5)	232 (38.8)
Not sure	142 (41.6)	95 (37.0)	237 (39.6)
Approve	33 (9.7)	94 (36.6)	127 (21.2)
Missing	2 (0.6)	0 (0.0)	2 (0.3)
Opinion of community/religious leader about getting the vaccine			
Disapprove	94 (27.6)	62 (24.1)	156 (26.1)
Not sure	168 (49.3)	114 (44.4)	282 (47.2)
Approve	77 (22.6)	81 (31.5)	158 (26.4)
Missing	2 (0.6)	0 (0.0)	2 (0.3)
Do you think your colleagues will get the vaccine?			
No	102 (29.9)	35 (13.6)	137 (22.9)
Not sure	169 (49.6)	115 (44.7)	284 (47.5)
Yes	68 (19.9)	107 (41.6)	175 (29.3)
Missing	2 (0.6)	0 (0.0)	2 (0.3)
Trust in the Ministry of Health			
Not at all	97 (28.4)	62 (24.1)	159 (26.6)
A little	118 (34.6)	87 (33.9)	205 (34.3)
Moderately	94 (27.6)	86 (33.5)	180 (30.1)
Very much	30 (8.8)	22 (8.6)	52 (8.7)
Missing	2 (0.6)	0 (0.0)	2 (0.3)

Abbreviations: COVID-19, coronavirus disease 2019; N, frequency.

### Factors associated with COVID-19 vaccine hesitancy

In total, 303 (50.7%; 95% CI 46.7 to 54.7%) participants were hesitant to receive the COVID-19 vaccine. The prevalence of COVID-19 vaccine hesitancy was significantly higher in Cameroon (56.9%; 95% CI 51.6 to 62.1%) than Nigeria (42.4%; 95% CI 36.5 to 48.6%). In Cameroon, vaccine hesitancy was more common in females than in males (63.6%; 95% CI 56.3 to 70.4% vs 49.4%; 95% CI 41.8 to 57.0%), while there was no gender difference in vaccine hesitancy among respondents from Nigeria (44.0%; 95% CI 36.4 to 51.8% vs 40.0%; 95% CI 30.9 to 49.9%).

**Figure 2.**
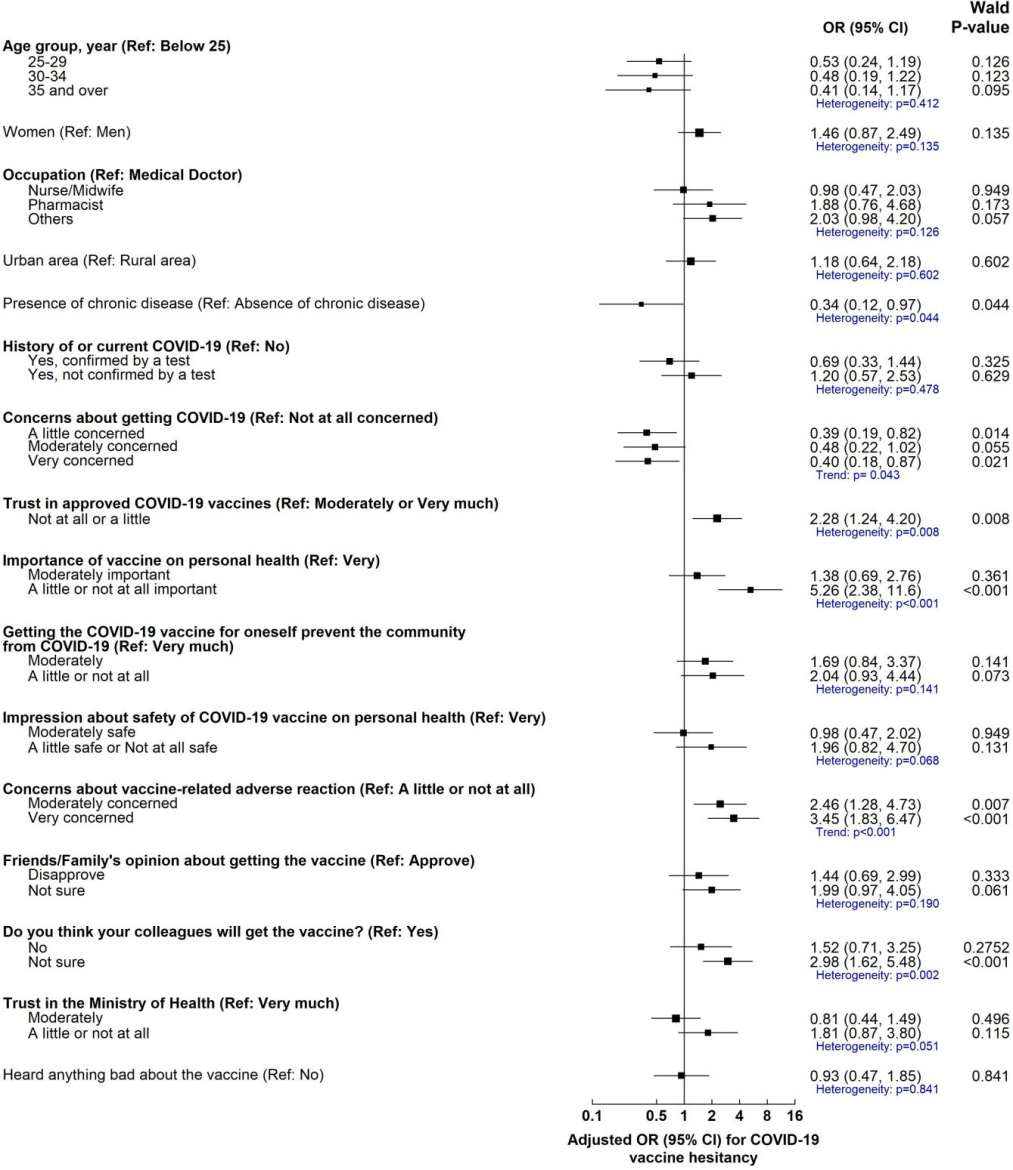
Factors associated with coronavirus disease 2019 (COVID-19) vaccine hesitancy on multivariable mixed-effect logistic regression analysis. Measures of associations are displayed as adjusted OR, black squares, with the 95% CI, horizontal spikes. The OR and 95% CI are plotted on the logarithmic scale. The solid black vertical line at OR of 1.0 refers to the null value. Statistical significance was based on the χ^2^ test for linear trend or heterogeneity, where applicable.

Table 4 summarizes the factors associated with COVID-19 vaccine hesitancy on univariate analysis. The intraclass correlation was 0.024. After adjusting for multiple confounders, participants with chronic disease had 66% lower odds (adjusted OR=0.34; 95% CI 0.12 to 0.97; p_heterogeneity_=0.044) of COVID-19 vaccine hesitancy than those with no history of chronic disease (Figure 2). In addition, participants who were very concerned about getting COVID-19 had 60% (0.39; 0.19 to 0.82; 0.043) lower odds of COVID-19 vaccine hesitancy than those with no concerns. Participants who had little or no trust in the approved COVID-19 vaccines had 2.3 times (2.28; 1.24 to 4.20; 0.008) higher odds of COVID-19 hesitancy compared with those with higher levels of trust. Participants who perceived COVID-19 vaccines to have little or no importance on their health were 5.3 times (5.26; 2.38 to 11.6; <0.001) more likely to be hesitant than those who perceived the vaccines as very important. Furthermore, those who were very concerned about COVID-19 vaccine-related adverse reactions were 3.5 times (3.45; 1.83 to 6.47; <0.001) more likely to be hesitant compared with those with little or no concerns. Moreover, those who were unsure whether their colleagues would get vaccinated had about threefold higher odds (2.98; 1.62 to 5.48; 0.002) of being hesitant than those who were sure their colleagues would receive the vaccine.

**Table 4.** Univariate mixed-effects logistic regression analysis of factors associated with COVID-19 vaccine hesitancy among healthcare workers in Cameroon and Nigeria (N=589)

Characteristics	Hesitant (N=303)	Not hesitant (N=295)	OR (95% CI)	p
Age group, y				0.0115^††^
<25	47	26	Reference	
25–29	172	152	0.69 (0.40 to 1.21)	0.198
30–34	54	73	0.41 (0.21 to 0.77)	0.006
≥35	30	44	0.46 (0.22 to 0.91)	0.026
Gender				0.045^††^
Male	121	152	Reference	
Women	182	152	1.40 (1.01 to 1.94)	0.045
Occupation				0.002^††^
Medical doctor	117	121	Reference	
Nurse/midwife	61	79	1.10 (0.69 to 1.75)	0.692
Pharmacist	35	24	2.77 (1.42 to 5.39)	0.003
Others	90	71	1.98 (1.24 to 3.17)	0.004
Area				0.345^††^
Rural	59	68	Reference	
Urban	244	227	1.19 (0.83 to 1.72)	0.345
Presence of chronic disease				0.016^††^
No	289	267	Reference	
Yes	14	28	0.44 (0.23 to 0.86)	0.016
History of or current COVID-19				0.008^††^
No	221	216	Reference	
Yes, confirmed by a test	36	51	0.54 (0.33 to 0.87)	0.012
Yes, not confirmed by a test	46	28	1.43 (0.86 to 2.39)	0.167
Treated poorly during the pandemic due to profession				0.143^††^
No	238	212	Reference	
Yes	65	83	0.76 (0.53 to 1.10)	0.143
Concerns about getting COVID-19				<0.001^†^
Not at all concerned	88	41	Reference	
A little concerned	91	69	0.64 (0.39 to 1.05)	0.078
Moderately concerned	68	76	0.47 (0.27 to 0.76)	0.003
Very concerned	56	109	0.27 (0.16 to 0.44)	<0.001
Concerned about family or friends getting COVID-19				<0.001^††^
Not at all concerned	51	29	Reference	
A little concerned	69	40	1.15 (0.62 to 2.14)	0.648
Moderately concerned	68	55	0.79 (0.43 to 1.44)	0.441
Very concerned	115	171	0.42 (0.24 to 0.72)	0.001
Concerned about patient getting COVID-19				<0.001^††^
Not at all concerned	45	31	Reference	
A little concerned	60	36	1.32 (0.73 to 2.36)	0.360
Moderately concerned	68	52	1.02 (0.58 to 1.77)	0.954
Very concerned	130	176	0.56 (0.35 to 0.90)	0.016
Trust in approved COVID-19 vaccines				<0.001^††^
Moderately or very much	65	225	Reference	
Not at all or a little	238	70	12.69 (8.50 to 18.94)	<0.001^††^
Importance of the vaccine on personal health				<0.001^††^
Very important	39	162	Reference	
Moderately important	57	97	2.38 (1.46 to 3.88)	0.001
A little or not at all important	207	36	22.78 (13.77 to 37.69)	<0.001
Getting the COVID-19 vaccine for oneself prevents the community from COVID-19				<0.001^††^
Very much	51	157	Reference	
Moderately	72	98	2.28 (1.45 to 3.56)	<0.001
A little or not at all	180	40	13.74 (8.54 to 22.10)	<0.001
Impression about safety of COVID-19 vaccine on personal health				<0.001^††^
Very safe	20	101	Reference	
Moderately safe	73	157	2.39 (1.35 to 4.24)	0.003
A little safe or Not at all safe	210	37	29.19 (15.76 to 54.07)	<0.001
Concerns about vaccine-related adverse reaction				<0.001^†^
A little or not at all	57	145	Reference	
Moderately concerned	64	76	2.19 (1.37 to 3.49)	0.001
Very concerned	182	74	6.73 (4.40 to 10.29)	<0.001
Confidence in answering vaccine-related questions				<0.001^††^
Very confident	89	140	Reference	
Moderately confident	95	105	1.34 (0.90 to 1.98)	0.150
A little or not at all confident	119	50	3.33 (2.16 to 5.13)	<0.001
Needs permission to take the vaccine				<0.001^††^
No	248	270	Reference	
Yes	55	25	2.56 (1.53 to 4.26)	<0.001
Friends and family's opinion about getting the vaccine				<0.001^††^
Approve	35	92	Reference	
Disapprove	134	98	2.88 (1.74 to 4.76)	<0.001
Not sure	134	105	2.78 (1.70 to 4.53)	<0.001
Opinion of community or religious leaders about getting the vaccine				0.028^††^
Approve	62	96	Reference	
Disapprove	81	75	1.45 (0.92 to 2.29)	0.112
Not sure	160	124	1.74 (1.16 to 2.61)	0.007
Do you think your colleagues will get the vaccine?				<0.001^††^
Yes	47	128	Reference	
No	77	60	2.93 (1.79 to 4.79)	<0.001
Not sure	179	107	4.04 (2.65 to 6.15)	<0.001
Trust in the MoH				<0.001^††^
Very much	64	170	Reference	
Moderately	110	95	2.97 (1.98 to 4.44)	<0.001
A little or not at all	129	30	11.10 (6.75 to 18.24)	<0.001
Heard anything bad about the vaccine				0.438^††^
No	39	64	Reference	
Yes	264	231	1.27 (0.69 to 2.32)	0.438

All p-values are generated from the Wald test unless reported otherwise.

^†^p-value for trend.

^††^p-values for heterogeneity unless stated otherwise.

Abbreviations: MoH, Ministry of Health; Reference, reference category.

## Discussion

Vaccine hesitancy remains a major obstacle, even among cohorts (such as HCWs) that are not particularly known to be reluctant to accept vaccines or other health interventions.^10^ This study evaluated the factors associated with COVID-19 vaccine hesitancy among HCWs in Cameroon and Nigeria. About 57% and 42% of HCWs in Cameroon and Nigeria, respectively, were hesitant to receive the COVID-19 vaccine. The presence of chronic disease and being concerned about getting COVID-19 were associated with lower odds of COVID-19 vaccine hesitancy. Lower levels of trust in the approved vaccines, perceived unimportance of the vaccine to personal health, concerns about COVID-19 vaccine-related adverse effects and uncertainties about colleagues getting the COVID-19 vaccines were associated with COVID-19 vaccine hesitancy.

Our estimates of vaccine hesitancy among HCWs were similar to those in other studies conducted in HCWs in Cameroon,^40^ Nigeria,^17^ Ghana,^21^ Togo,^15^ Ethiopia,^14,26^ Saudi Arabia^41^ and the UK.^42^ However, we observed a much higher proportion of COVID-19 vaccine hesitancy than in a previous report among HCWs in South Africa.^18^ This could be due to higher COVID-19–related mortality in South Africa than in Cameroon and Nigeria and effective vaccine promotion strategies. By contrast, the lower prevalence of hesitancy in this study than that reported in Congo (70%)^16^ could be because the latter study was conducted at an earlier period marked by higher levels of disinformation and conspiracy theories regarding COVID-19 vaccines.^16^ This period of disinformation was followed by intensive health promotion and education campaigns to address the myths and facts about COVID-19 vaccines. Ditekemena and colleagues^43^ showed that Congolese people of higher income levels were more willing to get immunized. Whether the relative economic situation of our participants influenced their vaccine-seeking behavior is beyond the scope of our study.

Similar to previous studies, participants concerned about COVID-19 vaccine-related adverse effects were more likely to be hesitant to receive the vaccine.^26,44,45^ In this same light, Agyekum and colleagues^20^ highlighted vaccine safety concerns being associated with vaccine hesitancy. In addition, we found that higher levels of mistrust in the approved COVID-19 vaccines were associated with higher odds of vaccine hesitancy. This overall mistrust in the approved vaccine's effectiveness, efficiency and side effects were highlighted by Botwe and colleagues in Ghana,^21^ and by Iliyasu and colleagues in Nigeria.^22^ Mistrust in the MoH and vaccine production and regulatory bodies have been associated with vaccine hesitancy.^23,46^ Whether this mistrust originates from conspiracies about COVID-19 and COVID-19 vaccines, lack of trust in pharmaceutical companies and national MoH, or the circumstances surrounding vaccine development, could not be fully answered in this study. We did not find evidence of an association between the level of trust in the MoH and vaccine hesitancy. This role of mistrust around the COVID-19 vaccine warrants further investigation using a more comprehensive qualitative study.

Higher levels of concern about getting COVID-19 were associated with lower odds of being vaccine-hesitant. In addition, those with chronic disease had lower odds of being vaccine-hesitant than those without any chronic disease. These findings are consistent with those of Angelo et al., among HCWs in Ethiopia,^27^ suggesting that those who perceive COVID-19 as a health threat are more cautious and likely to accept preventive measures. Furthermore, previous studies have reported lower odds of vaccine hesitancy with older age.^16,26,47^ Whether age is an independent determinant of COVID-19 vaccine hesitancy or whether this association is confounded by frailty, which increases with age, remains uncertain. However, we did not find evidence of an association between age and COVID-19 hesitancy after adjusting for multiple confounders, including a history of chronic disease. By contrast, lower levels of perception of the importance of COVID-19 vaccines to personal health was associated were higher odds of vaccine hesitancy, similar to a report from a previous survey among HCWs in Ethiopia.^23^ This study suggests that individuals who perceive COVID-19 as a threat, and vaccines to be beneficial, to their health are less likely to be hesitant.

This study indicates that colleagues’ vaccine acceptability is the most relevant social determinant of vaccine hesitancy among HCWs compared with religious and community leaders, family and friends. Participants who were unsure whether their colleagues would accept the COVID-19 vaccine were more likely to be hesitant to receive COVID-19 vaccines, similar to findings in HCWs in a previous report.^48^ However, we found no evidence that community or religious leaders, family and friends influenced participants’ decision to receive the COVID-19 vaccine. This is probably because community or religious leaders, families and friends depend on HCWs for health advice, and are, therefore, less likely to influence HCWs’ decisions on receiving the COVID-19 vaccine.

This study sheds more light on vaccine hesitancy and reiterated mistrust and safety concerns as a recurring factor associated with vaccine-hesitant behaviors. Despite the rising incidence of COVID-19 vis-à-vis vaccine mistrust, many might have adhered more to face masks than willingly opted for vaccines. As such, previously highlighted associated factors still need to be addressed to continually improve the COVID-19 vaccine's uptake. While this study provides insights into the factors associated with COVID-19 vaccine hesitancy in sub-Saharan Africa, some limitations are worth discussing. We could not verify participants’ location and occupation, which may lead to misrepresentation of participants. To mitigate this, the eligibility criteria for the study were clearly stated in the study's information sheet and questionnaire. In addition, the questionnaire included questions that permitted participants to state their current occupation and country of practice. Although the observed associations are internally valid, the findings from this study cannot be generalized to all HCWs in Cameroon and Nigeria because sampling was non-probabilistic. In addition, the over-representation of medical doctors in this study limits the generalizability of the findings. Furthermore, we cannot exclude the possibility of residual confounding and reverse causation. Finally, we acknowledge the possibility of selection bias as the study is more likely to include mostly HCWs who are more technology literate and with easier access to the internet, such as younger HCWs and those practicing in urban settings. Nevertheless, this study adds to the limited evidence on the determinants of COVID-19 vaccine hesitancy among HCWs in sub-Saharan Africa; previous studies were either qualitative or had limited adjustments for confounding. We estimated ORs with careful adjustment for confounders as recommended by the WHO. Careful adjustment for confounding is particularly important given the strong correlation between the determinants of COVID-19 vaccine hesitancy.

### Conclusions

This study highlights that COVID-19 vaccine hesitancy is high among HCWs in Cameroon and Nigeria. Concerns about vaccine-related side effects, lower perception of the importance of COVID-19 vaccines to personal health, mistrust of the approved vaccines and uncertainty about colleagues’ acceptability of the vaccine were associated with a higher likelihood of COVID-19 vaccine hesitancy. By contrast, participants who perceived COVID-19 as a threat to their health were less likely to be vaccine-hesitant. The relevance of this study indicates that targeted public health interventions addressing the factors associated with COVID-19 vaccine hesitancy could go a long way to improve COVID-19 vaccine uptake among HCWs. It is also pivotal to carry out qualitative studies to explore the concerns of these HCWs more profoundly.

## Data Availability

The data used to generate all results for this analysis are available from the corresponding author on reasonable request.
